# Macrophages, PPARs, and Cancer

**DOI:** 10.1155/2008/169414

**Published:** 2008-07-03

**Authors:** Jo A. Van Ginderachter, Kiavash Movahedi, Jan Van den Bossche, Patrick De Baetselier

**Affiliations:** ^1^Laboratory of Cellular and Molecular Immunology, Department of Molecular and Cellular Interactions, VIB, 1050 Brussels, Belgium; ^2^Laboratory of Cellular and Molecular Immunology, Vrije Universiteit Brussel, 1050 Brussels, Belgium

## Abstract

Mononuclear phagocytes often function as control switches of the immune system, securing the balance between pro- and anti-inflammatory reactions. For this purpose and depending on the activating stimuli, these cells can develop into different subsets: proinflammatory classically activated (M1) or anti-inflammatory alternatively activated (M2) macrophages. The expression of the nuclear peroxisome proliferator-activated receptors (PPARs) is regulated by M1- or M2-inducing stimuli, and these receptors are generally considered to counteract inflammatory M1 macrophages, while actively promoting M2 activation. This is of importance in a tumor context, where M1 are important initiators of inflammation-driven cancers. As a consequence, PPAR agonists are potentially usefull for inhibiting the early phases of tumorigenesis through their antagonistic effect on M1. In more established tumors, the macrophage phenotype is more diverse, making it more difficult to predict the outcome of PPAR agonism. Overall, in our view current knowledge provides a sound basis for the clinical evaluation of PPAR ligands as chemopreventive agents in chronic inflammation-associated cancer development, while cautioning against the unthoughtful application of these agents as cancer therapeutics.

## 1. INTRODUCTION

For many years, the centre of gravity in cancer research was focused on uncovering the activating (oncogenes) and/or deactivating (tumor suppressor genes) mutations in proliferating cells, causing these cells to adopt a cancerous phenotype [1]. By now, it has become increasingly clear that untransformed host cells, in particular cells of the immune system, are equally important in every aspect of cancer, from tumor initiation and progression to metastasis formation. Chronic inflammation, in response to microbial infections or persistent chemical insults, may provoke DNA damage in the surrounding tissue and induce cellular transformation [2–5]. Newly transformed cells can be eliminated or kept in a dormant state under the control of innate and adaptive immune cells, but ultimately the surviving “immunoedited” cancer cells are less immunogenic and more aggressive [6, 7]. Within the organoid context of a tumor, normal physiological functions of stromal cells—including a large fraction of leukocytes—are harnessed in favour of tumour progression, leading to modifications in the local extracellular matrix, neoangiogenesis, stimulation of cancer cell proliferation, and survival and promotion of cancer cell motility and invasiveness [8]. In each of these aspects of the tumor/immune interface, cells belonging to the mononuclear phagocyte system (including lineage committed bone marrow precursors, monocytes, and macrophages) have been implicated, functioning in different compartments (tumor site, periphery) and, mainly dictated by the context, having the potential to contribute to such diametrically opposed processes as tumor destruction or tumor promotion. The latter stresses the heterogeneity and polyvalency of this type of cells, making them indispensable for development, tissue homeostasis, inflammation, pathogen clearance, and wound healing [9]. As a consequence, drugs with the capacity of modifying macrophage activation are of potential interest in the treatment of different pathologies, including cancer. One such class of drugs is the ligands for peroxisome proliferator-activated receptors (PPARs), which are ligand-activated transcription factors belonging to the nuclear hormone receptor superfamily. The three PPAR isoforms (PPAR*α*, PPAR*β*/*δ*, and PPAR*γ*) are encoded by different genes and display differences in their tissue distribution, suggestive of specialized functions. Upon heterodimerization with retinoid X receptors (RXRs), PPARs bind to specific response elements (PPREs) in the promoter regions of a wide array of PPAR-regulated genes. As a consequence, PPARs have a broad range of effects on metabolism, cellular proliferation, differentiation, and immunity [10]. Of importance in the context of this review, each of the PPAR isoforms is expressed in cells of the monocyte/macrophage lineage and influences the phenotype of these cells [11–13]. This knowledge, in combination with the potential impact of monocytes/macrophages on tumor development, provides a rationale for investigating the modulation of PPAR activity in mononuclear phagocytes as therapeutic strategy in cancer research.

## 2. PLASTICITY oF MACROPHAGE ACTIVATION

### 2.1. The M1/M2 conceptual frame of macrophage activation

Macrophages belong to the most versatile cells of the body. Heterogeneity arises as macrophages differentiate from monocyte precursors and is determined by the genetic background as well as by specific tissue-related and immune-related stimuli [9, 14]. In this regard, microbial antigens, tumor products, immune complexes as well as Th1 or Th2 effector T cells and their secretory products influence the heterogeneity and the state of activation of macrophage populations [15, 16]. The better characterized response of macrophages to microbial molecules, cancer cells, and host cytokines is the release of inflammatory/microbicidal/tumoricidal products. This “classical activation” profile occurs in a type I cytokine environment (IFN-*γ*, TNF*α*) or upon recognition of pathogen-associated molecular patterns (LPS, lipoproteins, dsRNA, lipoteichoic acid, etc.) and endogenous “danger” signals (heat shock proteins, extracellular matrix components, HMGB1, etc.) [17]. As such, it plays an important role in protection against intracellular pathogens, and under certain conditions also cancer cells. Classically activated macrophages or M1 typically produce high levels of IL-12 and IL-23 [18] combined with low levels of IL-10 and are consequently strong promotors of Th1 immune responses. In addition, these cells exert antiproliferative and cytotoxic activities, resulting partly from their ability to secrete reactive nitrogen and oxygen species (NO, peroxynitrite, hydrogen peroxide, superoxide) and proinflammatory cytokines (TNF, IL-1, IL-6) [19–22]. Although such short-term inflammatory activity could be beneficial for the host in a tumor setting, the persistence of inflammatory processes often results in detrimental tissue and DNA damage contributing to cancer development [2–5]. Therefore, in the course of a response, inflammation is usually counteracted through the development of anti-inflammatory mechanisms. Ideally, this regulation must be spatially and temporally controlled.

Distinct mediators have been reported to inhibit the development of M1 and impart anti-inflammatory properties on macrophages, which were collectively termed “alternatively activated” or M2: Th2 cytokines, such as IL-4 and IL-13, deactivating cytokines, such as IL-10 and TGF-*β*, hormones, such as the glucocorticoids and vitamin D3, and apoptotic cells [23]. M2 have been reported to actively contribute to the pathology of helminth and protozoan infections, but also cancer [24–28]. The heterogeneity of these anti-inflammatory macrophages, whereby each stimulus induces both unique and overlapping gene expression repertoires, has urged the need for a more refined nomenclature. Gordon and colleagues proposed to restrict the definition of “alternative activation” to IL-4 and/or IL-13-elicited macrophages [29]. Subsequently, Mantovani and coworkers used a high production of IL-10 and low production of IL-12 as unifying theme for M2 [15]. Following this logic, a further subdivision was suggested between M2a, b, and c, representing IL-4/IL-13-stimulated (alternatively activated *sensu strictu*), immune complexes + TLR ligand-stimulated [30], and IL-10-stimulated (deactivated) macrophages, respectively. Though a usefull working scheme, it should be realised that any form of classification underscores the complexity of the in vivo situation, where macrophages are exposed to mixtures of stimuli and will adopt mixed functional profiles accordingly. This is exemplified by the determination of a consensus gene signature for in vivo induced M2 in different pathologies, which contains genes that are not inducible in vitro by any of the known M2 inducing stimuli [31].

### 2.2. Impact of PPARs on the macrophage activation state

All three isoforms of PPAR have been reported to be constitutively expressed in macrophages, with their mRNAs being upregulated during monocyte to macrophage differentiation [11, 32–34]. Though not all reports are in agreement, PPAR*γ*, but not PPAR*α* or -*β*/*δ*, may actually promote macrophage differentiation and contribute to the development of typical macrophage-associated features, such as phagocytosis of apoptotic cells [33–36]. The further regulation of PPARs in M1- or M2-conditioning environments has been thoroughly investigated in the case of PPAR*γ*. PPAR*γ* mRNA and protein are strongly induced in resident peritoneal macrophages and peripheral blood monocytes by the typical M2 inducers IL-4 and vitamin D3, suggesting a preferential association of high PPAR*γ* activity with M2 [33, 37]. Indeed, M1 stimuli such as IFN*γ*, LPS, TNF*α*, or IL-1*β* either have no effect on PPAR*γ* expression or were even inhibitory [37, 38]. On top of a higher level of PPAR*γ* receptors, M2 also produce more endogenous PPAR*γ* ligands, in part as a consequence of IL-4-mediated induction of 12/15 lipoxygenase [37, 39, 40]. This lipid-peroxidating enzyme generates the PPAR*γ* ligands 13-HODE and 15-HETE through the oxygenation of linoleic acid and arachidonic acid, respectively [40, 41]. In addition, both in mouse peritoneal macrophages and in human monocytes, IL-13 is able to increase the production and the nuclear localization of the PPAR*γ* ligand 15-deoxy-Δ^12,14^-prostaglandin J_2_ (15d-PGJ_2_) by a mechanism dependent on phospholipase A_2_ activation [42, 43].

Very few data are available on PPAR*α* gene regulation by pro- or anti-inflammatory stimuli, with only one study demonstrating a relatively unaltered PPAR*α* mRNA content in macrophages upon LPS treatment [44]. In general, systemic LPS treatment tends to decrease overall PPAR*α* expression levels, though it is unclear whether macrophages account for this phenomenon [45].

In the case of PPAR*β*/*δ*, current data suggest that this gene could be upregulated in both M1 and M2 polarizing conditions. On the one hand, PPAR*β*/*δ* mRNA is upregulated by LPS in macrophages, suggesting an association with M1 [44]. In keratinocytes, LPS and inflammatory cytokines not only induce PPAR*β*/*δ* gene transcription through an AP-1 site in the promoter, but also initiate the production of endogenous PPAR*β*/*δ* ligands [46, 47]. Of importance, the anti-inflammatory cytokine TGF-*β*1 counteracts PPAR*β*/*δ* expression in these cells [47]. This dynamic control of PPAR*β*/*δ* expression is particularly important in tissue injury and wound control [48]. If extendible to macrophages, these data would imply an enhanced PPAR*β*/*δ* transcriptional activity in an M1 context. On the other hand, in a very recent paper, IL-4 and IL-13 were shown to induce macrophage PPAR*β*/*δ* expression through a STAT6 binding site on its promoter [49]. Taken together, PPAR*β*/*δ* could be unique in its capacity to contribute to both M1 and M2 characteristics.

### 2.3. PPARs and M1 activation of macrophages

In macrophages, numerous inflammatory signalling pathways downstream of cytokine receptors or pattern recognition receptors orchestrate the inflammatory process. Central players in these signalling cascades are the NF-*κ*B, AP-1, and STAT family of transcription factors, whose binding sites can be found in the promoters of inflammatory cytokines, chemokines, metalloproteinases, iNOS, and other inflammatory genes [50, 51].

#### 2.3.1. PPAR*γ*

PPAR*γ* agonists dose-dependently inhibit the upregulation of inflammatory genes in macrophages in response to Toll-like receptor ligands and interferons. These effects are at least partially PPAR*γ*-dependent and can be attributed to an inhibition of NF-*κ*B, AP-1, and STAT1 transcriptional activities [52–54]. By now, the molecular machinery behind PPAR*γ*-mediated repression of NF-*κ*B-regulated genes has been uncovered and appears to depend on a mechanism termed ligand-dependent transrepression [55, 56]. Under steady-state conditions, some genes (e.g., iNOS) are occupied and actively repressed by the multisubunit NCoR repressor complex. Upon NF-*κ*B activation, the NCoR complex is degraded by the proteasome, NF-*κ*B p65-p50 heterodimers enter the nucleus, bind to NF-*κ*B elements in the promoter, and recruit coactivator complexes to initiate gene transcription. However, simultaneous ligand binding of PPAR*γ* leads to SUMOylation of a fraction of the PPAR*γ* molecules, which bind to NCoR and prevent its clearance from the promoter, leading to a sustained repressed state [57]. The requirement for the NCoR corepressor complex explains why only a subset of LPS-inducible genes is truely PPAR*γ*-regulated [54]. Remarkably, also AP-1-mediated gene transcription depends on the loss of NCoR complexes, suggesting a similar mechanism of PPAR*γ*-mediated repression of AP-1-regulated genes [58]. Of note, PPAR*γ* agonists such as 15d-PGJ_2_ and thiazolidinediones suppress a broader range of NF-*κ*B-regulated genes, irrespective of their NCoR dependence, and are even able to do so in PPAR null macrophages [54, 59]. In the case of 15d-PGJ_2_, this can be explained by a direct, PPAR*γ*-independent modification of critical cysteine residues in the I*κ*B kinase and the DNA-binding domains of NF-*κ*B subunits, inhibiting NF-*κ*B activity [60, 61].

The in vivo relevance of macrophage-expressed PPAR*γ* in attenuating inflammation has been demonstrated in macrophage-specific PPAR*γ* ^−/−^ animals. Unstimulated macrophages from these mice display an increased production of inflammatory mediators, indicating that endogenous PPAR*γ* ligands modulate macrophages under steady-state conditions. In addition, PPAR*γ* ^−/−^ macrophage recruitment to inflammatory sites is increased, and these macrophages overreact to inflammatory stimuli, resulting in increased severity of DSS-induced colitis [62]. Of importance, thiazolidinediones still improve colitis severity in colonic epithelium-specific PPAR*γ* ^−/−^ mice, but not in macrophage-specific PPAR*γ* ^−/−^ mice, suggesting that macrophages are the relevant targets of these compounds in this disease [63]. Also in models of insulin resistance and atherosclerosis, macrophage-specific PPAR*γ* was shown to inhibit inflammation and improve insulin sensitivity and reduce atherosclerotic lesion size, respectively [64, 65].

#### 2.3.2. PPAR*α*

PPAR*α* ligands are able to lower the secretion of inflammatory mediators in several cell types, including macrophages [66–70]. Similar to PPAR*γ*, PPAR*α* is able to transrepress NF-*κ*B and AP-1 transcriptional activity, though it does so in a different way. Inhibition of these transcription factors by PPAR*α* is independent of the promoter context but appears to depend on a physical interaction between PPAR*α* and the p65 Rel homology domain or the JNK-responsive part of c-Jun [71]. In addition, ligand-bound PPAR*α* transactivates the I*κ*B*α* promoter in a DNA binding-independent fashion, as such further attenuating NF-*κ*B activation [72]. Another parallel with PPAR*γ* is the importance of posttranslational modifications in the activity of PPAR*α*. Inflammatory stimuli such as LPS activate protein kinase C (PKC), which subsequently phosphorylates and inactivates PPAR*α*. However, statins inhibit PKC activation, increasing the pool of unphosphorylated transrepression-competent PPAR*α* which is entirely responsible for the anti-inflammatory activity of statins [73]. Also the well-characterised anti-inflammatory potential of glucocorticoids partially depends on PPAR*α*, possibly through a similar impact on PKC [74].

The in vivo significance of macrophage PPAR*α* is illustrated by enhanced atherosclerosis in low-density lipoprotein receptor-deficient mice transplanted with PPAR*α* ^−/−^ bone marrow, which is due to an increased inflammatory response of macrophages [75]. In the same vein, PPAR*α* ^−/−^ splenocytes produce significantly higher levels of inflammatory cytokines in aged mice, both under basal conditions or in the presence of LPS [76].

#### 2.3.3. PPAR*β*/*δ*

In contrast to PPAR*γ* and -*α*, PPAR*β*/*δ* can also be associated with M1 (besides M2) and may contribute to the proinflammatory phenotype of these macrophages. Indeed, under basal conditions PPAR*β*/*δ* ^−/−^ macrophages display a reduced expression of some (MCP-1, IL-1*β*, and MMP9), but not all (TNF*α*, IKK*β*) inflammatory mediators. As such, inflammation-driven atherosclerotic lesion formation is significantly reduced in PPAR*β*/*δ* ^−/−^ bone marrow chimeras. Mechanistically, PPAR*β*/*δ* forms a complex with the transcriptional repressor Bcl-6, preventing Bcl-6 from repressing inflammatory genes. However, upon synthetic ligand binding (e.g., GW501516) PPAR*β*/*δ* releases Bcl-6 and inflammation is dampened [77]. On top of that, PPAR*β*/*δ* activation induces the expression of mediators suppressing inflammatory cytokine/chemokine action (RGS, TIMP-3), altogether explaining the beneficial effects of PPAR*β*/*δ* agonists in inflammatory diseases such as atherosclerosis [78, 79].

### 2.4. PPARs and M2 activation of macrophages

PPARs not only antagonize M1 activation, but actually support M2 activation. Indeed, at least some of the reported anti-inflammatory effects of IL-4 or IL-13 are mediated through enhanced PPAR*γ* activity [80]. IL-4/IL-13 strongly increase the production of different endogenous PPAR*γ* ligands (13-HODE, 15-HETE, and 15d-PGJ_2_) and PPAR*γ* coactivators (PGC-1*β*), thereby stimulating the PPAR*γ* transactivating activity [37, 42, 43, 81]. As a matter of fact, some of the hallmark IL-4/IL-13-inducible M2 markers, such as MMR, arginase I, CD36, and dectin-1, depend on PPAR*γ* for full induction [42, 43, 82–84]. Following this logic, administration of PPAR*γ* ligands could be a valuable means of inducing M2 markers in vivo and altering macrophage functions [85]. The significance of these findings was recently established in macrophage-specific PPAR*γ* ^−/−^ mice [86]. Although LPS-induced release of IL-6 was not significantly different between w.t. and PPAR*γ* ^−/−^ macrophages, only in the PPAR*γ*-deficient cells was IL-4 unable to suppress IL-6, corroborating the notion that a subset of IL-4-dependent anti-inflammatory responses is regulated by PPAR*γ* [86]. These mice are defective in the in vivo generation of M2 to a similar extent as macrophage-specific IL-4R*α* ^−/−^ mice or STAT6 null mice. As a consequence, these mice are more resistant to Th2/M2-driven pathologies, such as cutaneous leishmaniasis.

Similar to PPAR*γ*, PPAR*β*/*δ* ablation was shown to diminish the M2 phenotype in macrophages, notably Kupffer cells and adipose tissue-resident macrophages, in vitro and in vivo (in PPAR*β*/*δ* ^−/−^ bone marrow chimeras or myeloid-specific PPAR*β*/*δ* ^−/−^ mice), and to increase inflammation. This results in systemic insulin resistance, increased adipocyte lipolysis, and hepatic dysfunction [49, 87].

Overall, it is clear from previous paragraphs that the regulation of PPARs by pro- or anti-inflammatory signals is one of the important factors that triggers macrophage polarization. It is however important to realize that the exact effects of PPARs on macrophages can depend on the source from which macrophages have been isolated (mouse versus human, different tissues, different pathogenic conditions, in vitro versus in vivo studies, etc.) and on the maturation stage of the macrophage population before PPAR activation.

## 3. M1 MACROPHAGES IN TUMOR INITIATION

**Figure 1:**
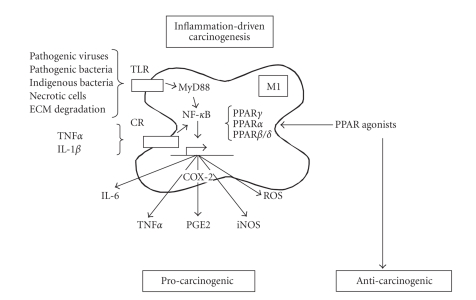
*Simplified scheme of the role of M1 macrophages in inflammation-driven carcinogenesis and the potential anticarcinogenic effect of PPAR ligands*. In the context of chronic pathogen infection or chemically induced chronic inflammation, exogenous and/or endogenous ligands for Toll-like receptors are present, which stimulate NF-*κ*B activation via the MyD88 pathway. Also inflammatory cytokines such as TNF*α* and IL-1*β* stimulate NF-*κ*B activatity through their respective cytokine receptors (CRs). Subsequently, NF-*κ*B transcribes a number of carcinogenic mediators, including IL-6, TNF*α*, COX-2, and iNOS amongst others. PPAR ligands are able to interfere with the induction of these inflammatory mediators, using different mechanisms. Activated PPAR*γ* tranrepresses NF-*κ*B activity, activated PPAR*α* physically interacts with NF-*κ*B, and activated PPAR*β*/*δ* unleashes the transcriptional repressor Bcl-6. Note that the anticarcinogenic actions of the PPAR agonists are only seen in inflammatory tumorigenesis but not in noninflammatory carcinogenesis.

Epidemiological studies clearly established a causal link between chronic inflammation—triggered by microbial infections or autoimmune diseases—and tumor development [2–5, 88]. Consequently, prolonged intake of nonsteroidal anti-inflammatory drugs has been proven to lower cancer incidence [89]. M1 macrophages are central orchestrators of the inflammatory response and are of critical importance in some of the well-known cancer-predisposing malignancies: *Helicobacter pylori* infection for gastric cancer [90], inflammatory bowel disease for colon carcinoma [91], and hepatitis for hepatocellular carcinoma [92]. Hence, inflammatory macrophages are actively involved in de novo carcinogenesis and the first steps of tumor development Figure 1.

### 3.1. Tumor-initiating role of NF-*κ*B in macrophages

The NF-*κ*B transcription factor is the master regulator of inflammation and has been shown to function as a tumor promoter in inflammation-associated cancers [50, 93]. NF-*κ*B can be activated both in cancer cells and immune cells, in particular M1 macrophages. The presence of such macrophages, bearing activated forms of NF-*κ*B and other inflammatory signaling molecules such as p38 MAPK, is seen in premalignant lesions (e.g., colonic polyps) [94]. Hence, it is of interest to gain insight into the relative importance of the NF-*κ*B cellular context (cancer cell versus macrophage) for carcinogenesis. A number of seminal papers have shed light on this issue in the past few years. In colitis-associated colon carcinoma formation, a prototypical example of inflammation-driven carcinogenesis, tumor formation, was reduced to the same extent in mice with either an enterocyte-specific or a myeloid cell-specific defect in the IKK*β*-dependent NF-*κ*B pathway. In the case of the myeloid cells, NF-*κ*B-mediated carcinogenesis depends on the production of inflammatory mediators that act as tumor-promoting paracrine factors [95]. In agreement with these findings, the absence of SIGIRR/TIR8, a negative regulator of NF-*κ*B, aggravates colitis-associated carcinogenesis. SIGIRR/TIR8 functions as a tumor suppressor both in colon epithelium and in bone marrow-derived cells [96]. Surprisingly, even in a model of noninflammatory tumor formation (DEN-induced hepatocarcinogenesis), NF-*κ*B activation in macrophages (Kupffer cells) appears to stimulate tumorigenesis through the secretion of hepatomitogens such as TNF*α* and IL-6 [97].

Apart from virally or bacterially induced cancers, how does NF-*κ*B get activated in macrophages during carcinogenesis? Recent findings demonstrate an important role for MyD88, the adaptor molecule in TLR and IL-1R signaling, in inflammation-associated or noninflammatory carcinogenesis alike [98–100]. Interestingly, functional polymorphisms in TLRs can predispose to certain types of carcinoma [101]. TLRs can become activated by endogenous ligands produced during cancer cell necrosis or extracellular matrix degradation, or—as shown in a transgenic model of gastric carcinogenesis—by the indigenous bacterial flora [102]. Another interesting pathway has been suggested by the Coussens lab, Calif, USA. Myeloid cells could become activated in response to immunoglobulins, putting the B cell-myeloid cell axis central in inflammation-associated carcinogenesis [103].

### 3.2. NF-*κ*B-regulated macrophage products responsible for tumor initiation

A large body of evidence points to inflammatory cytokines as major culprits for tumor stimulation. In the model of DEN-induced hepatocarcinogenesis, the estrogen-regulated difference in IL-6 production by male versus female Kupffer cells entirely accounts for the gender differences in tumor incidence [98]. While IL-6 is a hepatocyte mitogen, TNF*α* induces hepatocyte NF-*κ*B activation with a strong impact on tumorigenesis. Even under noninflammatory conditions, this carcinogenic TNF*α* is produced by endothelial cells and Kupffer cells [104]. In addition, carcinogen-stimulated chronic TNF*α* expression in liver inflammatory cells, presumably Kupffer cells, hyperactivates oval cells through TNF-R1, resulting in liver tumor formation [105]. Comparable mechanisms are at play in colitis-associated colon carcinoma, where macrophage-derived TNF*α* interacts with TNF-R1 in an autocrine way, creating an essential inflammatory loop for carcinogenesis [106]. One of the target genes of TNF*α*-stimulated NF-*κ*B in this model is COX-2 [107]. COX-2, via the production of PGE_2_, strongly promotes colon carcinogenesis [108]. Importantly, in premalignant lesions of both mice and humans, COX-2 is almost exclusively expressed in macrophages [108, 109]. Similarly, the NF-*κ*B target gene MMP9 is important for skin carcinogenesis and is exclusively produced by inflammatory cells [110]. Finally, other prototypical inflammatory macrophages products, such as nitric oxide and reactive oxygen species, have all been shown to contribute to oncogenesis [97, 111, 112].

### 3.3. Role of macrophage-specific PPARs in tumor initiation

Considering the importance of inflammatory macrophages as a trigger of carcinogenesis and the anti-inflammatory function of PPARs in macrophages, it seems logical to pursue PPAR ligation as a strategy to block the initial steps of tumor formation. Indeed, some of the most prominent tumor-promoting mediators of macrophages—TNF*α*, MMP9, iNOS—are known to be repressed by PPAR*γ* ligation [53, 113, 114]. In addition, PPAR*γ* ligands, which had no significant effect on tumor cell lines in vitro, were shown to exert potent inhibitory effects on tumors from the same cells in vivo, suggesting other targets besides cancer cells in the tumor-environment [115].

In line with this rationale, in vivo administration of PPAR*γ*, -*α*, and *β*/*δ* agonists invariably reduces tumor initiation in typical models of inflammation-associated carcinogenesis, such as colitis-driven colon carcinoma [116–118]. The situation is more blurred in colon cancer induced by genetic means (APC^Min^ mice) rather than by inflammatory stimulation, with contrasting reports describing tumor stimulation or repression upon PPAR*γ* ligation [119–121]. A recent study employed genetic means to assess the role of PPAR*γ* in chemically-induced (inflammatory) versus genetically-induced (noninflammatory) colon carcinogenesis. Haploinsufficiency of the PPAR*γ* gene promotes inflammatory carcinogenesis but has no effect in APC^Min^ mice [122]. Similarly opposing data exist on the role of PPAR*β*/*δ* in tumor formation in APC^Min^ mice, even between studies looking at APC^Min^ in a PPAR*β*/*δ* null background [123, 124].

Recent studies have studied transplantable tumor growth in PPAR*α* ^−/−^ or PPAR*β*/*δ* ^−/−^ mice. In both cases, tumor growth was strongly suppressed irrespective of the PPAR status of the cancer cells, indicating that host PPAR*α* and PPAR*β*/*δ* are important determinants in tumor formation [125, 126]. In the case of PPAR*α*, absence of the receptor resulted in overt inflammation and neutrophil-mediated tumor clearance [125]. Hence, the level of PPAR*α* stimulation might instruct the anti- or protumor activities of inflammatory cells: (i) absence of PPAR*α* leads to inflammatory cell-mediated tumor destruction, (ii) physiological levels of PPAR*α* stimulation could allow lower, protumoral levels of inflammation, and (iii) strong PPAR*α* stimulation with agonists could shutdown inflammation completely, prohibiting inflammation-driven carcinogenesis. Following this logic, scenarios (i) and (iii) reduce tumor growth, which has indeed been demonstrated experimentally [125, 127].

## 4. M1/M2 MACROPHAGES IN TUMOR PROGRESSION

Established tumors are often heavily infiltrated by leukocytes, of which tumor-associated macrophages (TAMs) can be a significant portion. The relevance of TAM in tumor biology is underscored by clinical studies showing a correlation between TAM abundance and poor prognosis, data which are particularly strong for breast, prostate, ovarian, and some types of lung cancers [128–130]. In addition, macrophage-deficient mice display reduced progression of tumors to a more malignant phenotype [131, 132]. TAMs are able to promote tumor progression via several mechanisms, including (i) induction of angiogenesis [133], (ii) remodelling of extracellular matrix [129], (iii) stimulation of cancer cell proliferation, migration, and invasion [134], and (iv) inhibition of adaptive immunity [135].

Current knowledge does not allow an unequivocal classification of TAM as prototypical M1 or M2 [28]. While TAMs are generally considered as anti-inflammatory M2, characterized by an IL-10^high^/IL-12^low^ cytokine profile and defective NF-*κ*B activation [27, 136, 137], these cells are also known to contribute to angiogenesis and cancer cell aggressiveness via the secretion of the M1-associated and NF-*κ*B-regulated mediators, such as TNF*α*, IL-1*β*, and MMP-9 [138–140]. The relative abundance of M1 or M2 markers in TAM could be related to the phase of tumor progression [141].

In any case, the relative plasticity and diversity of TAM make it difficult to predict the effect of PPAR ligation on these cells and on tumor outcome. In a mouse lymphoma model, we described an increased PPAR*γ* mRNA expression in M2-oriented TAM and splenic macrophages differentiated from a monocytic CD11b^+^Gr-1^+^ precursor [135, 142]. Remarkably, stimulation of TAM with PPAR*γ* ligands completely reverses TAM-mediated T-cell suppression, via an as yet unknown mechanism.

## 5. CONCLUDING REMARKS

In recent years, it has become clear that macrophages and other myeloid cells, such as mast cells and neutrophils, are central orchestrators of both tumor initiation and tumor progression. With the advent of the M1/M2 concept of macrophage activation, it has become clear that inflammatory M1 significantly participate in carcinogenic processes initiated by strong inflammatory stimuli, such as pathogens or certain chemicals. This finding opens a window of opportunity for the use of PPAR*γ*, -*α*, and *β*/*δ* agonists, some of which are already in clinical use for metabolic disorders, in chemoprevention of de novo tumor formation in patients at risk. However, the applicability of these compounds as anticancer agents is confounded by the often confusing findings in mice. In our view, confusion is the consequence of an insufficient insight in the participation of inflammatory cells in the models under study, making it difficult to extrapolate findings from one model to another. Overall, we feel that the usefulness of PPAR agonists is directly correlated with the extent to which inflammation is a driving force for carcinogenesis. Though this might hold true for the initial steps of tumor formation, the situation becomes more complicated in established tumors. Considering the plasticity and heterogeneity of tumor-associated macrophages, with a mixture of M1 and M2 markers and considerable differences between tumor types [28], it is more difficult to envisage a broad applicability of PPAR ligands for the modulation of TAM. However, treatment of certain typical macrophage-driven malignancies, such as breast carcinoma, could potentially benefit from these compounds.
